# Supplementary material for: End Sequence Analysis Toolkit (ESAT) expands the extractable information from single-cell RNA-seq data
Source: Genome Res. 2016 Oct;26(10):1397–410. doi: 10.1101/gr.207902.116 (PMC5052061; doi:10.1101/gr.207902.116)
Supplement: Supplemental Material [file supp_gr.207902.116_Supp_Materials.tar.gz › ESAT-esat_v0.1/doc/html/classumms_1_1core_1_1alignment_1_1_single_end_alignment-members.html]

ESAT: Member List


ESAT


- Main Page
- Related Pages
- Packages
- Classes
- Files

- Class List
- Class Index
- Class Hierarchy
- Class Members

All Classes Namespaces Files Functions Variables Enumerator Pages

- umms
- core
- alignment
- SingleEndAlignment

umms.core.alignment.SingleEndAlignment Member List

This is the complete list of members for umms.core.alignment.SingleEndAlignment, including all inherited members.

|  |  |  |
| --- | --- | --- |
| addBlocks(Annotation block) | umms.core.annotation.BasicAnnotation |  |
| addBlocks(Collection<?extends Annotation > blocks) | umms.core.annotation.BasicAnnotation |  |
| BasicAnnotation() | umms.core.annotation.BasicAnnotation |  |
| BasicAnnotation(SAMRecord samRecord) | umms.core.annotation.BasicAnnotation |  |
| BasicAnnotation(String ucsc) | umms.core.annotation.BasicAnnotation |  |
| BasicAnnotation(String referenceName, int start, int end, Strand orientation, String name) | umms.core.annotation.BasicAnnotation |  |
| BasicAnnotation(String referenceName, Strand orientation, String name, Collection<?extends Annotation > blocks) | umms.core.annotation.BasicAnnotation |  |
| BasicAnnotation(String referenceName, int start, int end) | umms.core.annotation.BasicAnnotation |  |
| BasicAnnotation(String referenceName, int start, int end, Strand orientation) | umms.core.annotation.BasicAnnotation |  |
| BasicAnnotation(String referenceName, int start, int end, String orientation) | umms.core.annotation.BasicAnnotation |  |
| BasicAnnotation(BasicAnnotation other) | umms.core.annotation.BasicAnnotation |  |
| BasicAnnotation(Annotation other) | umms.core.annotation.BasicAnnotation |  |
| BasicAnnotation(Collection<?extends Annotation > blocks) | umms.core.annotation.BasicAnnotation |  |
| BasicAnnotation(Collection<?extends Annotation > blocks, Strand orientation, String name) | umms.core.annotation.BasicAnnotation |  |
| BasicAnnotation(Collection<?extends Annotation > blocks, String name) | umms.core.annotation.BasicAnnotation |  |
| BasicAnnotation(String referenceName, CompoundInterval blocks, Strand orientation, String name) | umms.core.annotation.BasicAnnotation |  |
| BasicAnnotation(String referenceName, CompoundInterval blocks, Strand orientation) | umms.core.annotation.BasicAnnotation |  |
| BasicAnnotation(String referenceName, CompoundInterval blocks) | umms.core.annotation.BasicAnnotation |  |
| blocks | umms.core.annotation.BasicAnnotation | protected |
| compareTo(Annotation other) | umms.core.annotation.AbstractAnnotation |  |
| compareToAnnotation(Annotation b) | umms.core.annotation.AbstractAnnotation |  |
| compareToAnnotation(Annotation b, boolean useOrientation) | umms.core.annotation.AbstractAnnotation |  |
| complement() | umms.core.annotation.BasicAnnotation |  |
| contains(Annotation other) | umms.core.annotation.BasicAnnotation |  |
| copy() | umms.core.annotation.BasicAnnotation |  |
| createFromUCSC(String ucsc) | umms.core.annotation.BasicAnnotation | static |
| disect(Annotation a) | umms.core.annotation.BasicAnnotation |  |
| disect(List<?extends Annotation > disectors) | umms.core.annotation.BasicAnnotation |  |
| equals(Annotation other) | umms.core.alignment.SingleEndAlignment |  |
| umms::core::annotation::BasicAnnotation.equals(Annotation a, boolean useOrientation) | umms.core.annotation.AbstractAnnotation |  |
| expand(int deltaStart, int deltaEnd) | umms.core.annotation.AbstractAnnotation |  |
| fromFullInfoString(String fullInfoString) | umms.core.annotation.BasicAnnotation | static |
| fullyContains(Annotation other) | umms.core.annotation.AbstractAnnotation |  |
| getAlignmentEnd() | umms.core.alignment.SingleEndAlignment |  |
| getAlignmentStart() | umms.core.alignment.SingleEndAlignment |  |
| getAttribute(String string) | umms.core.alignment.SingleEndAlignment |  |
| getBlocks() | umms.core.annotation.BasicAnnotation |  |
| getBlocks(boolean oriented) | umms.core.annotation.BasicAnnotation |  |
| getChr() | umms.core.alignment.SingleEndAlignment |  |
| getChromosome() | umms.core.alignment.SingleEndAlignment |  |
| getCigarString() | umms.core.alignment.SingleEndAlignment |  |
| getDistanceTo(Annotation other) | umms.core.annotation.AbstractAnnotation |  |
| umms::core::annotation::BasicAnnotation.getEnd() | umms.core.annotation.BasicAnnotation |  |
| umms::core::alignment::Alignment.getEnd() | umms.core.alignment.Alignment |  |
| getFirstFragmentPositionStranded() | umms.core.alignment.SingleEndAlignment |  |
| getFlankingBlocks(Annotation spliceJunction) | umms.core.annotation.BasicAnnotation |  |
| getFragment(CoordinateSpace C) | umms.core.alignment.SingleEndAlignment |  |
| getFragmentEnd() | umms.core.alignment.SingleEndAlignment |  |
| getFragmentMidpoint(Annotation annot) | umms.core.alignment.SingleEndAlignment |  |
| getFragmentSize(CoordinateSpace C) | umms.core.alignment.SingleEndAlignment |  |
| getFragmentStart() | umms.core.alignment.SingleEndAlignment |  |
| getFragmentStrand() | umms.core.alignment.SingleEndAlignment |  |
| getFullInfoString() | umms.core.annotation.AbstractAnnotation |  |
| getHeader() | umms.core.alignment.SingleEndAlignment |  |
| getIntervalBetweenReads() | umms.core.alignment.SingleEndAlignment |  |
| getIsFirstMate() | umms.core.alignment.SingleEndAlignment |  |
| getLastFragmentPositionStranded() | umms.core.alignment.SingleEndAlignment |  |
| getLengthOnReference() | umms.core.annotation.AbstractAnnotation |  |
| getMappingQuality() | umms.core.alignment.SingleEndAlignment |  |
| getMidpoint() | umms.core.annotation.BasicAnnotation |  |
| getName() | umms.core.annotation.BasicAnnotation |  |
| getOrientation() | umms.core.annotation.BasicAnnotation |  |
| getOrientedEnd() | umms.core.annotation.AbstractAnnotation |  |
| getOrientedStart() | umms.core.annotation.AbstractAnnotation |  |
| getOverlap(Annotation other) | umms.core.annotation.BasicAnnotation |  |
| getPositionAtReferenceCoordinate(int referenceCoordinate, boolean ignoreOrientation) | umms.core.annotation.BasicAnnotation |  |
| umms::core::annotation::AbstractAnnotation.getPositionAtReferenceCoordinate(int referenceCoordinate) | umms.core.annotation.AbstractAnnotation |  |
| getReadAlignmentBlocks(CoordinateSpace C) | umms.core.alignment.SingleEndAlignment |  |
| getReadAlignments(CoordinateSpace space) | umms.core.alignment.SingleEndAlignment |  |
| getReadLength() | umms.core.alignment.SingleEndAlignment |  |
| getReadMates() | umms.core.alignment.SingleEndAlignment |  |
| getReadName() | umms.core.alignment.SingleEndAlignment |  |
| getReadSequence() | umms.core.alignment.SingleEndAlignment |  |
| getReferenceCoordinateAtPosition(int positionInAnnotation, boolean ignoreOrientation) | umms.core.annotation.BasicAnnotation |  |
| umms::core::annotation::AbstractAnnotation.getReferenceCoordinateAtPosition(int positionInAnnotation) | umms.core.annotation.AbstractAnnotation |  |
| getReferenceName() | umms.core.annotation.BasicAnnotation |  |
| getSAMEnd() | umms.core.annotation.AbstractAnnotation |  |
| getSAMStart() | umms.core.annotation.AbstractAnnotation |  |
| getScore() | umms.core.annotation.BasicAnnotation |  |
| getSize() | umms.core.annotation.AbstractAnnotation |  |
| getSpliceConnections() | umms.core.alignment.SingleEndAlignment |  |
| umms::core::annotation::BasicAnnotation.getStart() | umms.core.annotation.BasicAnnotation |  |
| umms::core::alignment::Alignment.getStart() | umms.core.alignment.Alignment |  |
| getStrand() | umms.core.annotation.AbstractAnnotation |  |
| getStrand(String orientation) | umms.core.annotation.AbstractAnnotation | static |
| getStrand(char orientation) | umms.core.annotation.AbstractAnnotation | static |
| getWeight() | umms.core.alignment.SingleEndAlignment |  |
| hasIndel() | umms.core.alignment.SingleEndAlignment |  |
| hasOrientation() | umms.core.annotation.AbstractAnnotation |  |
| intersect(Annotation other) | umms.core.annotation.BasicAnnotation |  |
| umms::core::annotation::AbstractAnnotation.intersect(List<?extends Annotation > others) | umms.core.annotation.AbstractAnnotation |  |
| isChimera() | umms.core.alignment.SingleEndAlignment |  |
| isDuplicate() | umms.core.alignment.SingleEndAlignment |  |
| isMapped() | umms.core.alignment.SingleEndAlignment |  |
| umms::core::annotation::BasicAnnotation.isNegativeStrand() | umms.core.annotation.AbstractAnnotation |  |
| umms::core::alignment::Alignment.isNegativeStrand() | umms.core.alignment.Alignment |  |
| isPaired() | umms.core.alignment.SingleEndAlignment |  |
| isProperPair() | umms.core.alignment.SingleEndAlignment |  |
| isUnoriented() | umms.core.annotation.BasicAnnotation |  |
| length() | umms.core.annotation.BasicAnnotation |  |
| main(String[] args) | umms.core.annotation.BasicAnnotation | static |
| MAX\_DISTANCE | umms.core.annotation.AbstractAnnotation | static |
| minus(Annotation other) | umms.core.annotation.BasicAnnotation |  |
| minus(Collection<?extends Annotation > others) | umms.core.annotation.BasicAnnotation |  |
| moveToCoordinate(int coordinateInReference) | umms.core.annotation.BasicAnnotation |  |
| numBlocks() | umms.core.annotation.BasicAnnotation |  |
| overlaps(Annotation other, int buffer, boolean considerOrientation) | umms.core.annotation.BasicAnnotation |  |
| overlaps(List<?extends Annotation > otherBlocks, int buffer) | umms.core.annotation.BasicAnnotation |  |
| umms::core::annotation::AbstractAnnotation.overlaps(Annotation other) | umms.core.annotation.AbstractAnnotation |  |
| umms::core::annotation::AbstractAnnotation.overlaps(Annotation other, int buffer) | umms.core.annotation.AbstractAnnotation |  |
| umms::core::annotation::AbstractAnnotation.overlaps(Annotation other, boolean considerOrientation) | umms.core.annotation.AbstractAnnotation |  |
| umms::core::annotation::AbstractAnnotation.overlaps(Collection<?extends Annotation > others) | umms.core.annotation.AbstractAnnotation |  |
| umms::core::annotation::AbstractAnnotation.overlaps(Collection<?extends Annotation > others, int buffer) | umms.core.annotation.AbstractAnnotation |  |
| overlapsStranded(Annotation other) | umms.core.annotation.AbstractAnnotation |  |
| setDuplicateFlag(boolean duplicateFlag) | umms.core.alignment.SingleEndAlignment |  |
| setEnd(int end) | umms.core.annotation.BasicAnnotation |  |
| setFragmentStrand(TranscriptionRead strand) | umms.core.alignment.SingleEndAlignment |  |
| setHasIndel(boolean h) | umms.core.alignment.SingleEndAlignment |  |
| setHeader(SAMFileHeader header) | umms.core.alignment.SingleEndAlignment |  |
| setIsFirstMate(boolean value) | umms.core.alignment.SingleEndAlignment |  |
| setName(String n) | umms.core.annotation.BasicAnnotation |  |
| setOrientation(Strand orientation) | umms.core.annotation.BasicAnnotation |  |
| umms::core::annotation::AbstractAnnotation.setOrientation(char orientation) | umms.core.annotation.AbstractAnnotation |  |
| setOrientedEnd(int orientedEnd) | umms.core.annotation.BasicAnnotation |  |
| setOrientedStart(int orientedStart) | umms.core.annotation.BasicAnnotation |  |
| setProperPairFlag(boolean properPairFlag) | umms.core.alignment.SingleEndAlignment |  |
| setReferenceName(String refName) | umms.core.annotation.BasicAnnotation |  |
| setScore(double s) | umms.core.annotation.BasicAnnotation |  |
| setSplicedEdges(Collection< Annotation > se) | umms.core.alignment.SingleEndAlignment |  |
| setStart(int start) | umms.core.annotation.BasicAnnotation |  |
| shift(int delta) | umms.core.annotation.BasicAnnotation |  |
| SingleEndAlignment(SAMRecord read) | umms.core.alignment.SingleEndAlignment |  |
| SingleEndAlignment(org.broad.igv.sam.Alignment read) | umms.core.alignment.SingleEndAlignment |  |
| SingleEndAlignment(SAMRecord read, boolean isFirstMateFlag) | umms.core.alignment.SingleEndAlignment |  |
| SingleEndAlignment() | umms.core.alignment.SingleEndAlignment |  |
| size() | umms.core.annotation.AbstractAnnotation |  |
| stitchTo(Annotation next) | umms.core.annotation.BasicAnnotation |  |
| toBED() | umms.core.annotation.AbstractAnnotation |  |
| toBED(int r, int g, int b) | umms.core.annotation.AbstractAnnotation |  |
| toBEDGraph() | umms.core.annotation.AbstractAnnotation |  |
| toCigar() | umms.core.annotation.AbstractAnnotation |  |
| toSAMRecord() | umms.core.alignment.SingleEndAlignment |  |
| toShortBED() | umms.core.annotation.AbstractAnnotation |  |
| toString() | umms.core.alignment.SingleEndAlignment |  |
| toUCSC() | umms.core.annotation.AbstractAnnotation |  |
| trim(int deltaStart, int deltaEnd) | umms.core.annotation.AbstractAnnotation |  |
| union(Annotation other) | umms.core.annotation.BasicAnnotation |  |


---

Generated on Fri Aug 15 2014 15:11:07 for ESAT by  

 1.8.7
